# Supplementary material for: Genomic analysis of family data reveals additional genetic effects on intelligence and personality
Source: Mol Psychiatry. 2018 Jan 10;23(12):2347–62. doi: 10.1038/s41380-017-0005-1 (PMC6294741; doi:10.1038/s41380-017-0005-1)
Supplement: Supplementary file 2 — Supplementary material Supplementary Tables 2 and 3 [file 41380_2017_5_MOESM2_ESM.docx]

Supplementary method.

Supplementary Figure 1.

To determine the number of ancestry components to control the log-Likelihoods and residual errors (square root of the residual variance) from linear regression models of the three traits on age, sex, and up to twenty principal components were examined. Based on these data, the optimal number of components to adjust for was seven for *g*, Years of Education, Digit Symbol Test, Vocabulary, and extraversion. Six were controlled for using logical memory, four for verbal fluency test, and nine were used for neuroticism.

Supplementary Table 2

Results of the variance components analysis for the individual tests of cognitive ability using the full model and the final model selected from a stepwise selection procedure.

| Phenotype | N | Model | Variance components | GRM_g_  h^2^ _g_ %(S.E) | GRM_kin_  h^2^ _kin_ % (S.E) | SRM_Family_  e_f_^2^ % (S.E) | SRM_Sibling_  e_s_^2^ % (S.E) | SRM_Couple_  e_c_^2^ % (S.E) |
| --- | --- | --- | --- | --- | --- | --- | --- | --- |
| Cognitive |  |  |  |  |  |  |  |  |
| Vocabulary | 19 269 | Full | GKFSC | 23.4 (2.0) | 39.4 (4.7) | 1.0×10^−4^ (2.2) | 6.5 (1.3) | 30.7 (2.6) |
|  | 19 269 | Selected | GKSC | 25.6 (2.0) | 30.1 (2.8) |  | 7.3 (1.3) | 27.4 (1.9) |
|  |  |  |  |  |  |  |  |  |
| Verbal Fluency | 19 380 | Full | GKFSC | 18.3 (2.0) | 30.7 (5.2) | 1.0×10^−4^ (2.4) | 4.5 (1.3) | 16.2 (3.1) |
|  | 19 380 | Selected | GKSC | 18.9 (2.1) | 27.1 (2.9) |  | 4.6 (1.3) | 14.7 (2.1) |
|  |  |  |  |  |  |  |  |  |
| Digit Symbol Test | 19 385 | Full | GKFSC | 20.2 (2.1) | 22.7 (5.1) | 1.0×10^−4^ (2.4) | 8.0 (1.4) | 17.3 (3.1) |
|  | 19 385 | Selected | GKSC | 21.4 (2.1) | 14.7 (2.8) |  | 8.1 (1.3) | 13.2 (2.3) |
|  |  |  |  |  |  |  |  |  |
| Logical Memory | 19 365 | Full | GKFSC | 11.4 (2.0) | 24.0 (5.2) | 1.0×10^−4^ (2.5) | 5.1 (1.4) | 5.3 (3.2) |
|  | 19 365 | Selected | GKS | 11.9 (2.0) | 20.3 (2.8) |  | 5.4 (1.4) |  |

|  |  |  | Minor allele frequency (MAF) | | | | | |  |  |
| --- | --- | --- | --- | --- | --- | --- | --- | --- | --- | --- |
| Phenotype | *N* |  | 0.001-0.01  h^2^ %(S.E) | > 0.01-0.1  h^2^ %(S.E) | > 0.1-0.02  h^2^ %(S.E) | > 0.2-0.3  h^2^ %(S.E) | > 0.3-0.4  h^2^ %(S.E) | > 0.4-0.5  h^2^ %(S.E) |  | Total variance explained  h^2^ %(S.E) |
| Number of SNPs |  |  | 3 898 626 | 3 320 146 | 1 413 929 | 1 061 603 | 930 841 | 872 346 |  | 11 497 491 |
| Cognitive |  |  |  |  |  |  |  |  |  |  |
|  |  |  |  |  |  |  |  |  |  |  |
| Vocabulary | 7 102 |  | 13.8 (9.2) | 0.7 (4.9) | 7.7 (3.5) | 5.1 (3.4) | 12.2 (3.3) | 5.1 (2.9) |  | 44.5 (9.6) |
|  |  |  |  |  |  |  |  |  |  |  |
| Verbal Fluency | 7 144 |  | 23.1 (9.3) | 2.3 (5.2) | 7.1 (3.5) | 0.1 (3.2) | 6.2 (3.1) | 2.6 (2.7) |  | 41.4 (9.9) |
|  |  |  |  |  |  |  |  |  |  |  |
| Digit Symbol Test | 7 152 |  | 6.2 (9.0) | 4.4 (5.1) | 1.0×10^−4^ (3.4) | 3.7 (3.3) | 3.3 (3.1) | 4.5 (2.7) |  | 22.1 (9.6) |
|  |  |  |  |  |  |  |  |  |  |  |
| Logical Memory | 7 148 |  | 11.4 (9.3) | 1.2 (5.1) | 0.8 (3.4) | 3.3 (3.2) | 2.0 (3.1) | 3.0 (2.6) |  | 21.7 (9.8) |
|  |  |  |  |  |  |  |  |  |  |  |

Supplementary Table 3.

GREML-MS analysis of the four cognitive tests used in the construction of the general intelligence phenotype using six Minor Allele Frequency cut offs.
